# Safety and efficacy of a novel biodegradable occluder- Pansy ® occluder compared with traditional nondegradable occluder in the closure of patent foramen ovale

**DOI:** 10.3389/fcvm.2026.1704287

**Published:** 2026-01-27

**Authors:** Bingyi Li, Haowei Zeng, Hang Xie, Beidi Lan, Yushun Zhang

**Affiliations:** 1 Department of Cardiovascular Surgery, The First Affiliated Hospital of Xi'an Jiaotong University, Xi'an, China; 2 Department of Cardiovascular Medicine, The First Affiliated Hospital of Xi'an Jiaotong University, Xi'an, China

**Keywords:** biodegradable occluder, nondegradable occluder, patent foramen ovale, propensity score matching, transcatheter closure

## Abstract

**Background:**

Traditional nondegradable patent foramen ovale (PFO) occluders pose mechanical complications and may limit potential transseptal access, necessitating biodegradable alternatives without permanent implants. The safety and efficacy of the fully biodegradable Pansy® occluder have not been directly compared with traditional nondegradable devices.

**Objects:**

To compare the Pansy® occluder with traditional nondegradable occluders for percutaneous closure of PFO, focusing on complications, adverse events, and closure outcomes during follow-up.

**Methods:**

We retrospectively analyzed consecutive patients who underwent PFO closure with the Pansy® occluder (*n* = 44) or traditional nondegradable occluders (*n* = 381, predominantly Amplatzer occluder) between June 2019 and June 2020 at the First Affiliated Hospital of Xi'an Jiaotong University. Propensity score matching (1:2) was performed for age, gender, spontaneous right-to-left shunt (RLS) grade, and provokable RLS grade. Outcomes included procedural success, complications, adverse events, effective closure rate, and residual shunt at 6 and 12 months.

**Results:**

Before matching, Pansy® recipients were younger and included fewer female patients. After matching, baseline characteristics were well balanced. Procedural success was 100% in both groups, with no severe adverse events. Device-related thrombosis occurred in three patients in the Pansy® group (6.82% vs. 0.00%, *P* = 0.01); all cases resolved after 2–3 months of rivaroxaban without embolic complications. This difference may be influenced by more frequent scheduled TEE surveillance in the Pansy® group. Effective closure rates at 6 and 12 months were comparable between groups (88.64% vs. 87.50%, *P* = 0.85; 95.45% vs. 92.31%, *P* = 0.77), with no significant differences in residual shunt.

**Conclusions:**

The Pansy® occluder demonstrated comparable safety and efficacy compared to nondegradable devices for PFO closure.

## 1 Introduction

The Amplazter patent foramen ovale (PFO) occluder remains the most widely used device for PFO closure, although other approved occluders demonstrate comparable safety and efficacy. Current occluders predominantly consist of nondegradable metallic and synthetic fabric materials. Although PFO closure is generally effective, reported complications include cardiac perforation (1), erosion, chronic inflammation (2, 3), arrhythmias (4), and thrombus formation (5). These implants may restrict future transseptal access to the left atrium, potentially complicating subsequent interventions for left heart disease, including percutaneous repair or replacement of the heart valve [transcatheter mitral valve replacement, and transcatheter edge-to-edge repair (MitraClip and PASCAL)], arrhythmia ablation, pulmonary vein isolation, or left atrial appendage closure. Biodegradable occluders may offer advantages for patients with nickel hypersensitivity or nickel allergy by reducing nickel ion release. In addition, their potentially lower thrombogenicity, more rapid endothelialisation, and controlled degradation of the bioabsorbable membrane may be beneficial in patients with haematological concerns (6). Collectively, these considerations highlight the need for next-generation occluders that provide effective closure without permanent implantation.

The Pansy ® occluder consists of a biodegradable polydioxanone single filament and a polyethylene terephthalate matrix membrane. The polydioxanone component preserves structural integrity and mechanical strength during the early post-implantation period and gradually degrades into water and carbon dioxide. Transesophageal echocardiography (TEE) at 6 and 12 months demonstrated substantial reductions in both disc dimensions and overall occluder thickness (7, 8). Prior studies have reported a favorable safety profile and high complete closure rates with Pansy ® occluder (7–10). However, comparative studies evaluating the Pansy® occluder against traditional nondegradable devices remain limited.

In this study, we compared biodegradable and nondegradable occluders in patients undergoing percutaneous closure of PFO, evaluating complications, adverse events, and closure rates during follow-up. These data provide insights into the long-term safety and efficacy of biodegradable closure devices, informing clinical decision-making and future device development.

## 2 Methods

### 2.1 Study design and patient population

We retrospectively analyzed consecutive patients who underwent PFO closure using either the Pansy® occluder or traditional nondegradable occluders (primarily Amplatzer devices) at the First Affiliated Hospital of Xi'an Jiaotong University between June 2019 and June 2020. PFO was diagnosed using standardized contrast transthoracic echocardiography (cTTE), demonstrating spontaneous or provoked right-to-left shunt (RLS) after injection of 10 mL agitated saline via an antecubital vein. Indications for PFO closure included cryptogenic stroke, migraine, transient ischemic attack (TIA), or syncope. Cryptogenic stroke was defined as ischemic stroke without an identifiable cause other than a high-risk PFO with RLS. Ischemic stroke was characterized by acute focal neurological deficits presumed ischemic in origin, persisting ≥24 h or accompanied by corresponding infarction on brain MRI (11). Exclusion criteria were: (a) age <16 years; (b) incomplete clinical data; (c) follow-up duration <6 months; (d) absence of 6 months cTTE results.

### 2.2 Study device and procedure

**Figure 1:**
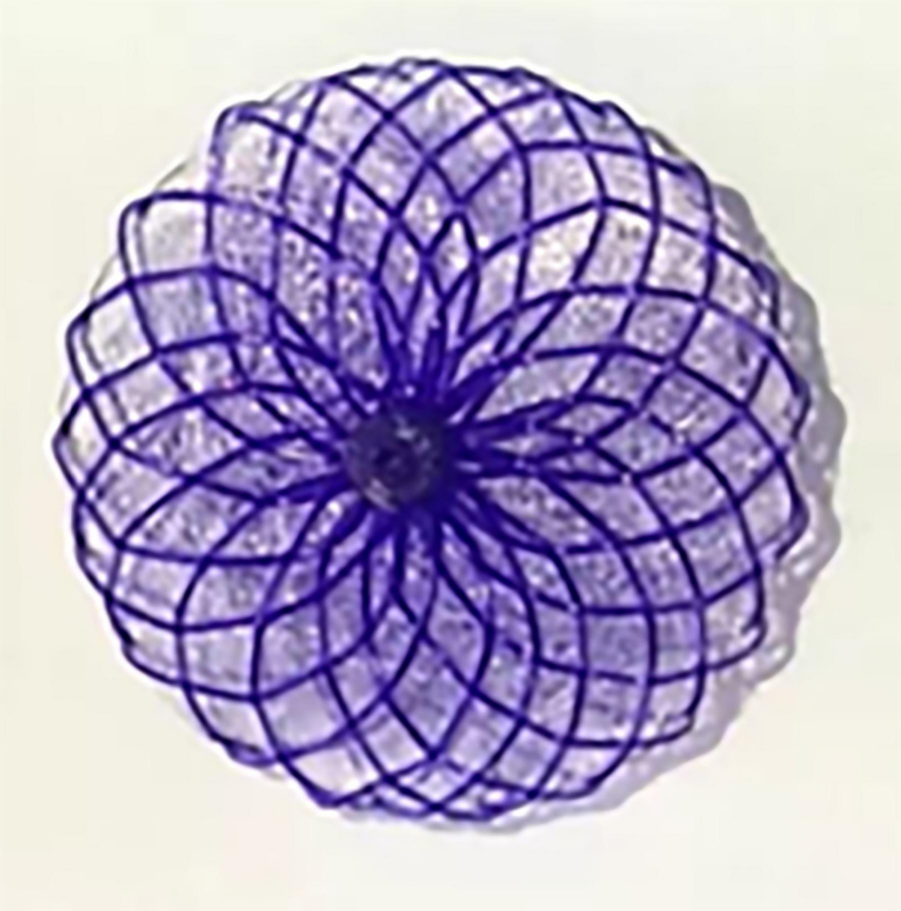
The pansy® occluder.

The Pansy ® occluder (Figure 1), developed by Shanghai China JinKui Medical Occluder Device Company, comprises a biodegradable polydioxanone single filament and a polyethylene terephthalate matrix membrane. The polydioxanone component provides structural integrity and mechanical strength and gradually degrades into water and carbon dioxide. The polyethylene terephthalate matrix membrane facilitates sealing of residual leaks, consistent with the mechanism of Amplatzer occluders. The device is available in seven sizes:18/18 mm, 24/18 mm, 24/24 mm, 30/24 mm, 30/30 mm, 34/24, 34/34 mm, allowing selection of different right- and left-atrial disc sizes.

The traditional nondegradable occluders included Amplatzer PFO occluders (*n* = 225), Amplatzer atrial septal defect (ASD) occluders (*n* = 90) and Cardi-O-Fix PFO occluders (*n* = 66). The Institutional Review Board of the First Affiliated Hospital of Xi'an Jiaotong University approved the study (No: XJTU1AF2015LSL-049). All patients provided written informed consent for the procedure and for the use of anonymized clinical data for research. PFO closure was performed in accordance with current guideline recommendations. Patients received pretreatment with aspirin (3–5 mg/kg) and clopidogrel (50–75 mg) within 48 h before the intervention. Under local anesthesia (1% lidocaine in the right groin area), all procedures were guided by fluoroscopy and transthoracic echocardiography (TTE). Venous access was obtained via the right femoral vein. A multipurpose catheter was advanced to cross the PFO, after which the delivery sheath was positioned. Unfractionated heparin (80–100 IU/kg) was administered after venous access. The occluder was advanced into the left atrium through the PFO using an 11-F transseptal sheath and delivery catheter. After confirming device stability, the left disc (umbrella) was deployed within the left atrium, followed by gentle retraction of the device and sheath to appose the atrial septum. The right disc was then released by withdrawing the sheath. Proper device position and stability were confirmed by TTE. Contrast injection through the delivery catheter was performed to assess residual RLS before and after device release. After the procedure, patients were prescribed aspirin (100 mg once daily for 6 months) and clopidogrel (75 mg once daily for 3 months).

### 2.3 Endpoints and definitions

Procedure success was defined as successful implantation in catheterization without procedure complications. Severe adverse events include pericardial tamponade, embolism, device embolism, and severe bleeding. Device embolism was defined as displacement of the occluder from the atrial septum to any other anatomical location. RLS severity was graded on a 0–3 scale based on the maximum number of microbubbles observed per frame in the left atrium after contrast injection, either at rest or during the Valsalva maneuver: grade 0, no microbubbles; grade 1, <10 microbubbles; grade 2, 10–30 microbubbles; and grade 3, >30 microbubbles. Effective closure at 6 or 12 months was defined as residual RLS grade 0 or 1 on cTTE. Residual shunt was defined as grade 2 (moderate residual shunt) or grade 3 (large residual shunt) on follow-up imaging.

### 2.4 Follow-up

Patients underwent routine follow-up at 1, 3, 6, and 12 months after the procedure. TTE and electrocardiography were performed at 1 and 3 months. TTE was used to evaluate occluder positioning and detect pericardial effusion. At 6 and 12 months, both TTE and cTTE were performed; TEE was performed at the operator's discretion to evaluate device size changes and thrombus formation. cTTE was used to assess residual shunts. Follow-up data were collected during outpatient's visits, and all patients completed at least 6 months of follow-up.

### 2.5 Statistics analysis

To improve comparability of baseline characteristics between groups, clinical outcomes were analyzed using propensity score matching (PSM). The propensity score represents the conditional probability of specific exposures given baseline covariates. Four clinical variables were included in the analysis: age, gender, spontaneous RLS grade, and provokable RLS grade. Nearest neighbor matching was performed at a 1:2 ratio with a caliper width of 0.2. Continuous variables with a normal distribution are presented as mean ± SD and were compared using two-tailed unpaired *t*-tests. Non-normally distributed continuous variables are presented as median (IQR) and were analyzed with Mann–Whitney *U*-tests. Categorical variables are presented as counts (percentages) and were compared using *χ*^2^ or Fisher's exact tests. Statistical significance was set at *P* < 0.05 (two-sided). PSM analysis was conducted using EmpowerStats (R-based), and all other statistical analyses were performed with SPSS 23.

## 3 Results

### 3.1 Basic characteristics of two groups

**Figure 2:**
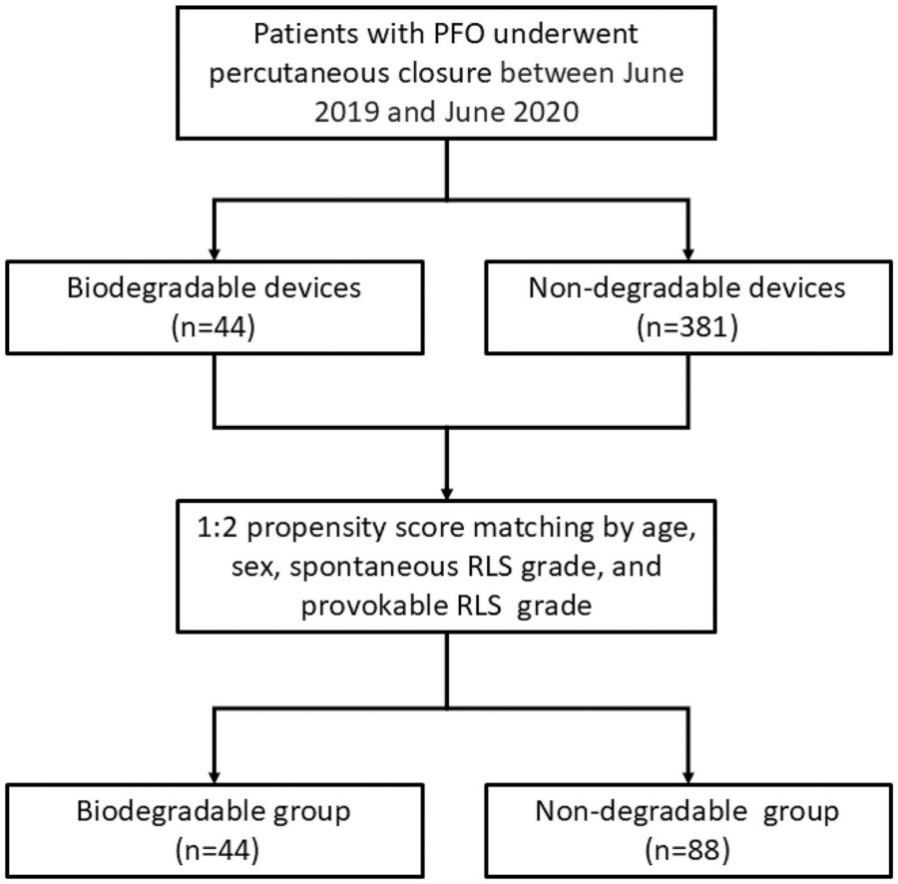
Flow chart of the study. PFO: patent foramen ovale; RLS: Right-to-left shunt.

During the study period, 44 patients underwent PFO closure with the Pansy® occluder, and 381 patients received traditional nondegradable occluders. Baseline characteristics of the two cohorts are summarized in Table 1. Patients in the Pansy® group were younger and included fewer female patients. After PSM (1:2), 44 patients in the Pansy® group were matched to 88 controls (Figure 2). Nondegradable occluders included Amplatzer PFO occluders (*n* = 51), Amplatzer ASD occluders (*n* = 22), and Cardi-O-Fix PFO occluders (*n* = 15). Baseline characteristics were well balanced after matching with no significant differences in age, gender distribution, closure indications (migraine, cryptogenic stroke, TIA, syncope), comorbidities (hypertension, diabetes, atherosclerosis, coronary artery disease), spontaneous RLS grade, or provokable RLS grade. Procedural success was achieved in 100% of cases in both groups.

**Table 1:** Basic characteristics.

Characteristic	Before matching			After matching		
	Biodegradable （*n* = 44)	Traditional （*n* = 381)	*P*-value	Biodegradable （*n* = 44)	Traditional （*n* = 88)	*P*-value
Age	36.59 ± 11.23	41.27 ± 13.78	0.03	40.53 ± 14.73	36.59 ± 11.23	0.1206
Female	24 (54.55%)	274 (72.11%)	0.016	24 (54.55%)	47 (53.40%)	1
Migraine	27 (61.36%)	233 (61.15%)	0.979	27 (61.36%)	50 (56.82%)	0.7550
Syncope	2 (4.55%)	34 (8.92%)	0.323	2 (4.55%)	4 (4.54%)	1
Stroke	6 (13.64%)	33 (8.66%)	0.279	6 (13.64%)	4 (4.54%)	0.1306
TIA	2 (4.55%)	25 (6.56%)	0.604	2 (4.55%)	4 (4.54%)	1
Arrhythmias	2 (4.55%)	6 (1.57%)	0.17	2 (4.55%)	2 (2.27%)	0.8575
Hypertension	8 (18.18%)	51 (13.39%)	0.384	8 (18.18%)	13 (14.77%)	0.8007
Diabetes	1 (2.27%)	6 (1.57%)	0.731	1 (2.27%)	1 (1.14%)	1
Atherosclerosis	2 (4.55%)	26 (6.82%)	0.564	2 (4.55%)	9 (10.22%)	0.4358
Coronary heart disease	0 (0.00%)	12 (3.15%)	0.232	0 (0.00%)	3 (3.41%)	–
Spontaneous RLS			0.169			0.8225
0	23 (52.27%)	183 (48.28%)		23 (52.3)	48 (54.5)	
1	13 (29.55%)	75 (19.79%)		13 (29.5)	20 (22.7)	
2	4 (9.09%)	39 (10.29%)		4 (9.1)	9 (10.2)	
3	4 (9.09%)	82 (21.64%)		4 (9.1)	11 (12.5)	
Provokable RLS			0.554			-
0	0 (0.00%)	0 (0.00%)		0 (0.00%)	0 (0.00%)	
1	0 (0.00%)	0 (0.00%)		0 (0.00%)	0 (0.00%)	
2	0 (0.00%)	3 (0.79%)		0 (0.00%)	0 (0.00%)	
3	44 (100.00%)	376 (99.21%)		44 (100%)	88 (100%)	
Success of the intervention	381 (100%)	44 (100%)	–	44 (100%)	88(100%)	–

TIA, transient ischemic attack; RLS, right-to-left shunt.

### 3.2 Adverse events of two groups

Adverse events of two groups are showed in Table 2. No severe adverse events—including pericardial tamponade, embolism, device embolism, severe bleeding, or death—occurred in either group. However, the Pansy® occluder group had a significant higher incidence of device-related thrombosis than nondegradable group (6.82% vs. 0.00%, *P* = 0.01), with three cases identified. The first case involved a 45-year-old woman in whom thrombus was detected by TEE at 6 months after she discontinued aspirin and clopidogrel prematurely due to minor ecchymosis. The second case occurred in a 42-year-old woman and was identified by TTE at 3 months. The third case involved a 38-year-old woman and was detected by TEE at 6 months. All three patients were treated with rivaroxaban for two to three months, resulting in thrombus resolution without embolic complications confirmed by TEE.

**Table 2:** Adverse events of percutaneous closure of PFO.

Group	Pansy ® （*n* = 44)	Nondegradable （*n* = 88)	*P*-value
Pericardial tamponade	0 (0.00%)	0 (0.00%)	–
Embolism	0 (0.00%)	0 (0.00%)	–
Device embolism	0 (0.00%)	0 (0.00%)	–
Severe bleeding	0 (0.00%)	0 (0.00%)	–
Device-related thrombosis	3 (6.82%)	0 (0.00%)	0.01
Chest tightness	0 (0.00%)	0 (0.00%)	–
Pericardial effusion	0 (0.00%)	0 (0.00%)	–
Episodes of atrial fibrillation	0 (0.00%)	2 (2.27%)	0.80
Supraventricular arrhythmia	1 (2.27%)	3 (3.41%)	0.86
Palpitation	1 (2.27%)	0 (0.00%)	0.72
Cough	1 (2.27%)	0 (0.00%)	0.72
All-cause death	0 (0.00%)	0 (0.00%)	–
PFO-related death	0(0.00%)	0(0.00%)	–

PFO, patent foramen ovale.

### 3.3 Effective closure rate at 6 months after the procedure

At 6 months follow-up, all matched patients were evaluated for procedural efficacy (Table 3). The effective closure rates did not differ significantly between groups (88.64% vs. 87.50%, *P* = 0.85). Medium residual shunt (grade 2) occurred in 4.55% and 3.41% (*P* = 0.87) of patients, and large shunt (grade 3) occurred in 6.82% and 9.01% (*P* = 0.91) in the Pansy® and nondegradable group, respectively. Overall, there were no significant differences between two groups in effective closure rate or residual shunt at 6 months after the procedure.

**Table 3:** Effective closure rate at 6 months after the procedure.

Group	Pansy ® (*n* = 44)	Nondegradable (*n* = 88)	*P*-value
Effective closure	39 (88.64%)	77 (87.50%)	0.85
Medium residual shunt	2 (4.55%)	3 (3.41%)	0.87
Large residual shunt	3 (6.82%)	8 (9.01%)	0.91

### 3.4 Effective closure rate at 12 months after the procedure

At 12 months follow-up, ten patients in the nondegradable group were lost to follow-up, therefore, 44 patients in the Pansy® occluder group and 78 in the nondegradable group were evaluated for closure efficacy (Table 4). The effective closure rates did not differ significantly between groups (95.45% vs. 92.31%, *P* = 0.77). Medium residual shunt (grade 2) was observed in 0.00% vs. 2.56% (*P* = 0.74) of patients, and large residual shunt (grade 3) occurred in 4.55% vs. 5.13% (*P* = 0.77) in the Pansy® and nondegradable group, respectively. Overall, no significant differences were observed between two groups in effective closure rate or residual shunt at 12 months after the procedure.

**Table 4:** Effective closure rate at 12 months after the procedure.

Group	Pansy ®（*n* = 44)	Nondegradable （*n* = 78)	*P*-value
Effective closure	42 (95.45%)	72 (92.31%)	0.77
Medium residual shunt	0 (0.00%)	2 (2.56%)	0.74
Large residual shunt	2 (4.55%)	4 (5.13%)	0.77

## 4 Discussion

In this retrospective, propensity-matched cohort study, both the Pansy® occluder and nondegradable occluders achieved 100% procedural success without severe adverse events. At 6 and 12 months, effective closure rate and residual shunt were comparable between the two groups. Notably, device-related thrombosis was observed more frequently in the Pansy® group.

For biodegradable occluders, the degradation kinetics must be carefully controlled to maintain sufficient mechanical support until endothelialization is complete. Polydioxanone-based devices are designed to degrade into benign metabolites within approximately 6 months, which broadly align with expected endothelialization timelines. Prior imaging assessments at 6 and 12 months have demonstrated substantial reductions in disc dimensions and overall device thickness (7, 8). However, complete degradation timelines and their clinical implications require confirmation through longer follow-up in larger cohorts.

Biodegradable occluders have evolved from partially bioabsorbable designs to fully bioabsorbable designs, aiming to reduce complications attributable to permanent metallic implants while preserving the possibility of future transseptal access. The BioSTAR device, a partially bioabsorbable PFO occluder with a metal framework and a porcine collagen membrane, demonstrated low complication and embolic recurrence rates, but relatively high rates of mild-to-medium residual shunt were reported at 6 months and 2 years follow-ups (12–18). Late complications remain clinically significant (19). The point pressure from radially expanding nitinol struts on the adjacent structures, primarily the atrial roof or aorta, is the most likely driving force for cardiac perforation and tamponade (20). In the present study, the Pansy® occluder comprises a biodegradable polydioxanone monofilament framework and a polyethylene terephthalate matrix membrane. The polydioxanone scaffold provides early mechanical stability and is expected to degrade into biocompatible byproducts, whereas the polyethylene terephthalate membrane maintains shunt sealing, mirroring the mechanism of Amplatzer devices. Complete degradation eliminates residual metallic components, thereby mitigating perforation and erosion risks. Nonetheless, extended follow-up remains necessary to evaluate delayed complications.

Previous studies reported an incidence of device-related thrombosis of approximately 1.8%–2.5% (5, 21), primarily in the CardoSEAL group (5, 22–24). In our cohort, thrombosis was detected more frequently in the Pansy® group. A plausible explanation is differential imaging intensity: most patients in the Pansy® group underwent scheduled TEE at 6 and 12 months to monitor device size changes, whereas TEE in the nondegradable group was typically performed only when clinically indicated (e.g., significant residual shunt or recurrent neurologic events). Because TEE is the gold standard for visualisation of cardiac and aortic structures and sources of embolism as well as semi-quantitative assessment of the shunt (25), more intensive surveillance may have increased thrombus detection in the Pansy® group. Although no definitive evidence currently links biodegradable occluders to higher thrombosis risk, surface changes during degradation could theoretically delay endothelialization and facilitate thrombus formation. This hypothesis is consistent with observations of increased thrombosis risk reported in other biodegradable implants (26). Further studies are required to clarify whether thrombosis risk differs by device type.

We observed no significant between -group differences in effective closure rate or residual shunt at either 6 or 12 months. The BioSTAR device showed relatively high rates of mild-to-medium residual shunt at 6 months and 2 years follow-ups (12–18). While some studies report comparable closure rates between BioSTAR and Amplatzer occluders beyond 6 months (27). The rate of residual shunt is a metric to measure the success of PFO closure. The device is fully protective only when it is completely endothelialized. Residual shunt is clinically meaningful because medium to large residual shunt is associated with increased risk of recurrent stroke or TIA (28). Absence of residual shunt at 6 months after closure is associated with improvement in migraine burden by >50% (29). Additionally, in patients undergoing PFO closure because of decompression sickness, three out of four patients who experienced decompression sickness recurrence had a residual shunt (30). According to Butera and colleagues, residual shunts were classified into two types: tunnel-like or between disk defect and accessory defect next to device rim or accessory defect (31). Early residual shunt is commonly attributed to incomplete endothelialization, whereas resting shunt (32), larger device size (33), atrial septal aneurysm (32–34), larger left atrial opening (35), and large right atrial opening (35) have been associated with residual flow. Late residual shunt can be attributed to special anatomy, inappropriate device selection, or missed diagnosis of other abnormalities (such as ASD or pulmonary arteriovenous fistula). In patients with large residual shunt, the second implantation of a device was effective in many cases (31, 33, 36–38).

We declare several limitations. First, the retrospective, non-randomized design introduces potential selection bias. Second, the single-center setting and relatively small sample size—particularly in the biodegradable device group— limit generalizability. Third, the lack of systematic TEE monitoring—particularly in the nondegradable device cohort—an important methodological limitation and may have led to under-detection of device-related thrombus or other findings.

Overall, clinical adoption of the Pansy® occluder appears feasible, but multi-center studies with standardized imaging follow-up and longer observation are needed to confirm degradation kinetics and long-term safety outcomes.

## 5 Conclusion

In this retrospective, propensity-matched cohort study, percutaneous PFO closure using the biodegradable Pansy® occluder demonstrated procedural success and follow-up efficacy comparable to traditional nondegradable occluders through 12 months. Further multicenter studies with longer follow-up and uniform imaging protocols are warranted to evaluate complete degradation, late complications, and device-related thrombosis risk.

## Data Availability

The raw data supporting the conclusions of this article will be made available by the authors, without undue reservation.
